# Editor's Letter-Box

**Published:** 1900-10-20

**Authors:** 


### Editor's Letter-Box.

[Our Correspondents are reminded that prolixity is a great bar to publication, and that brevity of style and conciseness of statement greatly facilitate early insertion.]

A. B. writes: Your article on Modern Sociology contains an error that should be corrected. It states that in "Denmark only *girls* are whipped." This is wrong; girls are whipped with the birch in Norway, also in Sweden, up to 15 and 18 years of age. In the Russian peasant courts girls are whipped, and in our own colony of South Australia. This information can be tested, as regards Norway, Sweden, and South Australia, in the Appendix to the Government

Report of British Industrial and Reformatories Inquiry for 1896.

[We have submitted the above to the writer of the article referred to, who informs us that in a paper on the Treatment of Juvenile Offenders (Howard Medal Prize Essay), by Rosa M. Barrett, which was read before the Royal Statistical Society last March, the author says: "Denmark is the only country where whipping may be given to girls under 12." Apparently A. B. has been able to obtain fuller information on the subject.—Ed. Hospital.]

